# Radionuclide-fluorescence Reporter Gene Imaging to Track Tumor Progression in Rodent Tumor Models

**DOI:** 10.3791/57088

**Published:** 2018-03-13

**Authors:** Alessia Volpe, Francis Man, Lindsay Lim, Alex Khoshnevisan, Julia Blower, Philip J. Blower, Gilbert O. Fruhwirth

**Affiliations:** ^1^Department of Imaging Chemistry and Biology, School of Biomedical Engineering and Imaging Sciences, King's College London

**Keywords:** Cancer Research, Issue 133, Cell tracking, reporter gene imaging, sodium iodide symporter, fluorescent protein, positron emission tomography, [^18^F]tetrafluoroborate, fluorescence microscopy, viral gene delivery

## Abstract

Metastasis is responsible for most cancer deaths. Despite extensive research, the mechanistic understanding of the complex processes governing metastasis remains incomplete. *In vivo* models are paramount for metastasis research, but require refinement. Tracking spontaneous metastasis by non-invasive *in vivo* imaging is now possible, but remains challenging as it requires long-time observation and high sensitivity. We describe a longitudinal combined radionuclide and fluorescence whole-body *in vivo* imaging approach for tracking tumor progression and spontaneous metastasis. This reporter gene methodology employs the sodium iodide symporter (NIS) fused to a fluorescent protein (FP). Cancer cells are engineered to stably express NIS-FP followed by selection based on fluorescence-activated cell sorting. Corresponding tumor models are established in mice. NIS-FP expressing cancer cells are tracked non-invasively *in vivo* at the whole-body level by positron emission tomography (PET) using the NIS radiotracer [^18^F]BF_4_^-^. PET is currently the most sensitive *in vivo* imaging technology available at this scale and enables reliable and absolute quantification. Current methods either rely on large cohorts of animals that are euthanized for metastasis assessment at varying time points, or rely on barely quantifiable 2D imaging. The advantages of the described method are: (i) highly sensitive non-invasive *in vivo* 3D PET imaging and quantification, (ii) automated PET tracer production, (iii) a significant reduction in required animal numbers due to repeat imaging options, (iv) the acquisition of paired data from subsequent imaging sessions providing better statistical data, and (v) the intrinsic option for *ex vivo* confirmation of cancer cells in tissues by fluorescence microscopy or cytometry. In this protocol, we describe all steps required for routine NIS-FP-afforded non-invasive *in vivo* cancer cell tracking using PET/CT and *ex vivo* confirmation of *in vivo* results. This protocol has applications beyond cancer research whenever *in vivo* localization, expansion and long-time monitoring of a cell population is of interest.

**Figure:**
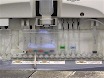


## Introduction

Metastatic disease is the cause for most cancer-related deaths 1. Despite extensive research into metastatic processes, reliable monitoring of cancer metastasis in animal model systems is difficult to achieve. Recent advances in whole-body imaging technologies and multi-modal imaging approaches have enabled non-invasive *in vivo* cell tracking^2^,^3^,^4^,^5^. The latter can be used as a tool to monitor the presence, distribution, quantity, and viability of cells, non-invasively and repeatedly in a live animal or a human.

The purpose of the method described here is to longitudinally and non-invasively track cancer cells in 3D in living rodent tumor models. Using this method, researchers will be able to accurately quantify tumor progression including metastatic spread in 3D. Compared to traditional non-imaging-based techniques, this method offers the acquisition of quantitative data with largely reduced animal numbers. Another feature of this method is that it allows correlation of *in vivo* imaging with streamlined downstream *ex vivo* analysis of the tracked cells in harvested tissues by histology or cytometry^3^,^6^.

The rationale for the development of this method was to provide an *in vivo* tool for the monitoring and quantification of the whole metastatic process in rodent tumor models. Importantly, it was designed to minimize animal use while at the same time reducing inter-animal variability. Longitudinal non-invasive whole-body imaging is excellently suited to inform on metastatic outgrowth, for which *per se* it is difficult to predict accurately the time and location of its occurrence. Whole-body 3D imaging has therefore been at the center of method development. To close the scale gap between whole-body *in vivo* imaging and potential downstream *ex vivo* histological confirmation, a multi-scale imaging approach based on a dual-mode radionuclide-fluorescence reporter was adopted^3^,^6^.

Positron emission tomography (PET) is the most sensitive 3D whole-body imaging technology currently available offering excellent depth penetration and absolute quantification^7^ with a resolution of <1 mm^8^,^9^. Currently, preclinical cell tracking on the whole-body level by radionuclide imaging can reliably detect cells at densities of ~1,000 cells per volume of a million cells^3^,^6^ with resolutions in the sub-millimeter region. Unlike intravital microscopy, it cannot detect single spreading cancer cells, but it does not require surgical procedures (*e.g.* window chambers), is not limited to a small field of view, and not by low tissue penetration and scatter. Bioluminescence imaging provides an inexpensive alternative, but is associated with scatter and light absorption issues as well as poor depth penetration, and consequently severely limited in quantification^2^. Fluorescence whole-body imaging has been used for the acquisition of 3D images, but it is much less sensitive compared to bioluminescence or radionuclide technologies^2^. Nonetheless, fluorescence offers the opportunity to perform *ex vivo* downstream tissue analysis by cytometry or microscopy. The latter closes the scale-gap between macroscopic whole-body imaging (mm resolution) and fluorescence microscopic tissue analysis (µm resolution)^3^. Therefore, radionuclide and fluorescence modalities complement each other, ranging from the whole-body level to the (sub-)cellular scale.

Reporter gene imaging is ideally suited for prolonged cell tracking as required in metastasis research. In this application it is superior to direct cell labeling as it is (i) not affected by label dilution and thus not limited in tracking time, and (ii) better reflects live cell numbers. Consequently, whole-body cell tracking is particularly useful for applications in which traceable cells proliferate or expand *in vivo*, for example in cancer research^3^,^6^, for the detection of teratoma formation in stem cell research, or for the quantification of immune cell expansion^5^.

Various radionuclide-based reporter genes are available^2^. These include enzymes such as the herpes simplex virus HSV11 thymidine kinase (HSV1-*tk*), transporters such as the sodium iodide symporter (NIS) or the norepinephrine transporter (NET), as well as cell surface receptors such as the dopamine D2 receptor (D2R). NIS is a glycosylated trans-membrane protein that actively mediates iodide uptake, for example in follicular cells in the thyroid gland for the subsequent synthesis of thyroid hormones^10^. This process is driven by the symport of Na^+^ and relies on the cellular sodium gradient, which is maintained by the Na^+^/K^+^-ATPase^11^. Consequently, NIS better reflects living cell numbers than other reporters as iodide/radiotracer uptake is linked to an active Na^+^/K^+^ gradient rather than the mere presence of the transporter. Traditionally, radioiodide has been used for NIS imaging. For cell tracking, alternative NIS radiotracers that are not metabolically entrapped in the thyroid have been reported to be superior^6^. This recently developed PET radiotracer [^18^F]tetrafluoroborate ([^18^F]BF_4_^-^)^12^,^13^ shows superior pharmacokinetics as compared to radioiodide^6^ while being available at high specific activities^14^ without the need for complex radiochemistry facilities. [^18^F]BF_4_^-^ can be synthesized via two different ways. The first method is based on isotope exchange of non-radioactive ^19^F in BF_4_^-^ with radioactive ^18^F^12^. The second method is through addition of ^18^F to non-radioactive boron trifluoride^14^. The latter method was reported to yield higher specific activities^14^ and is the method of choice for preclinical imaging.

NIS is highly expressed in the thyroid tissues. It is also expressed in the salivary, lachrymal and lactating mammary glands as well as the stomach, but at lower levels as compared to the thyroid gland^10^. Therefore, excellent contrast imaging in other body regions can be achieved using NIS. It is also highly homologous between human, rat and mouse^10^. Moreover, there are no reports of toxicity upon ectopic NIS expression in non-thyroidal cells. Importantly, NIS has also not been associated with host immune responses, neither in humans nor in rodents. NIS has been used as a reporter gene to measure promoter activity^15^,^16^,^17^ and gene expression^18^,^19^,^20^,^21^,^22^,^23^ in several different contexts. It has also been used for non-invasive imaging of gene therapy vectors^24^,^25^, and to track cells in cardiac^4^, hematopoietic^26^, inflammation^5^, and neural studies^27^. Recently, NIS has also been used as a reporter gene to track cancer metastasis *in vivo*^3^,^6^.

In summary, the main advantages of this method over previous techniques are: (i) highly sensitive non-invasive 3D *in vivo* localization and quantification of metastatic spread, (ii) automated production of [^18^F]BF_4_^-^ at high molar activities, (iii) a significant reduction in required animals through longitudinal imaging, (iv) the acquisition of paired data from subsequent imaging sessions resulting in improved statistical data, which in turn further reduces animal use, and (v) the intrinsic option for *ex vivo* confirmation of cancer cells in tissues by cytometry or fluorescence microscopy.

## Protocol

This protocol meets all requirements set by United Kingdom (UK) legislation and the local Ethical Review panel. When following this protocol, ensure the procedures also meet all requirements dictated by national legislation and local Ethical Review panel. Ensure every experiment involving radioactivity is compliant with legislation and local rules and performed safely.

### 1. Engineering and characterization of cancer cells to express the radionuclide-fluorescence fusion reporter NIS-FP

NOTE: For simplicity, mEGFP A206K is abbreviated as "GFP", and mCherry as "RFP" in the subsequent sections of this protocol.

1. Generation of lentiviral particles
  1. To produce lentivirus particles, co-transfect 293T cells with the following four plasmids using a suitable transfection method: (i) the reporter gene encoding plasmid (pLNT SFFV NIS-GFP or pLNT SFFV NIS-RFP (see Supplementary Information), (ii) the third-generation lentiviral packaging plasmids pRRE and (iii) pRSV-Rev, and (iv) a virus envelope containing plasmid, *e.g*, pMD2.G. Pre-mix plasmids before adding them to the transfection mix. Perform transfection in a cell culture hood. NOTE: Additional transfection information is provided in Supplementary Information.
  2. Assess transfection success after 48 h by standard wide-field fluorescence microscopy with filter settings appropriate for the chosen fusion reporter (NIS-GFP or NIS-RFP). NOTE: Fluorescence signals are indicative of reporter gene transfection and therefore only a surrogate for successful co-transfection, not an indicator of successful virus production.
  3. Harvest the virus particle-containing supernatant using a syringe and remove floating cells and cell debris by filtering through a 0.45 µm sterile polyethersulfone (PES) filter. Transfer to a sterile 1.5 mL polypropylene reaction tube. Perform virus work in a cell culture hood and ensure no live virus leaves the contained environment.
2. Transduction and selection of NIS-FP expressing cancer cell lines
  1. Use pure fresh virus from step 1.1.3 mixed 1:1 (v/v) with the optimal growth medium of each cancer cell line (DMEM for MDA-MB-231 cells and RPMI 1640 for 4T1 cells; see Materials Table for media composition). Perform transduction in a cell culture hood. NOTE: A more general protocol is referred to in Supplementary Information.
  2. Transduce cancer cells with virus-containing medium in an incubator with humidified atmosphere containing 5% (v/v) CO_2_ at 37 °C for 72 h (work on 6-well or 12-well scale using 1 mL or 0.4 mL of the virus mixture from step 1.2.1).
  3. Monitor target cell NIS-FP expression by fluorescence microscopy.
  4. Expand successfully transduced cells to a scale of 3-10 million cells using standard culture conditions (see ATCC for MDA-MB-231 and 4T1 cell lines).
  5. Use fluorescence-activated cell sorting (FACS) to purify NIS-FP expressing cells from non-transduced cells. NOTE: FACS can be a source of mycoplasma infections; it is recommended to check for mycoplasma before virus production and transduction but also after FACS and before step 1.3.
3. Characterization NIS-FP expressing cancer cell lines
  1. Confirm reporter expression by standard flow cytometry as described elsewhere^28^.
  2. Confirm reporter integrity by standard immunoblotting as described elsewhere^29^.
  3. Analyze intracellular fusion reporter localization by confocal fluorescence microscopy. NOTE: Staining with or co-expression of a plasma membrane marker^6^ and subsequent co-localization analysis^30^ will facilitate this step.
  4. Analyze radiotracer uptake in NIS-FP expressing cell lines. NOTE: Any radioactive NIS substrate compatible with existing equipment is suitable, *e.g.* the iodide isotopes (^123^I^-^, ^124^I^-^, ^125^I^-^, ^131^I^-^), ^99m^TcO_4_^-^, ^188^ReO_4_^-^, [^18^F]SO_3_F^-^ or [^18^F]BF_4_^-^.
    1. Seed 10^6^ purified cells in 6-well plates in their optimal growth medium (see Table of Materials) one day prior to the experiment. Prepare all samples in triplicate and include control samples: (i) "specificity controls", *i.e.* NIS-FP expressing cells pre-incubated with a competitive NIS substrate to test uptake specificity; (ii) "parental cell controls", *i.e.* cells not expressing NIS-FP but receiving radiotracer to test basal uptake in parental cells.
    2. On the next morning, wash cells once with serum-free growth medium.
    3. Incubate the cells in serum-free growth medium in the presence of 50 kBq ^99m^TcO_4_^-^ or [^18^F]BF_4_^-^ for 30 min at 37 °C (1 mL total volume). For specificity controls, pre-incubate the cells for 30 min with the competitive substrate NaClO_4_^-^ (12.5 µM final concentration). Keep the competitive substrate concentration constant throughout the experiment.
    4. Collect the supernatant and transfer 100 µL to a prepared collection tube labeled "supernatant".
    5. Wash the cells twice with 1 mL ice-cold phosphate-buffered saline (PBS) containing Ca^2+^/Mg^2+^. Collect each wash solution and transfer 100 µL of each into a prepared collection tube labeled "wash1" or "wash2", respectively.
    6. Lift the cells by adding 500 µL PBS containing 0.25 % (w/v) trypsin and 0.53 mM EDTA, and incubating at 37 °C until the cells detach (check visually using a microscope). Transfer the suspension into a prepared collection tube labeled "cells". Wash the wells with 500 µL ice-cold PBS containing Ca^2+^/Mg^2+^ and add to the tube "cells". Pellet the cells by centrifugation (250 x g, 4 min, 4 °C).
    7. Count all four sample types from each well using a gamma counter appropriately set for the radioisotope of choice (here: ^99m^Tc or ^18^F). NOTE: Due to the large number of samples in this assay, it is recommended to use an automated γ-counter capable of automatic decay correction.
    8. Analyze data by summing obtained gamma counts of samples from each well to determine a total radioactivity count per well. NOTE: take aliquoting in steps 1.3.4.4 and 1.3.4.5 into account by multiplying numbers by 10 for "supernatant", and "wash" fractions.
    9. Express cellular radiotracer uptake as %Uptake as indicated in Equ.1. Calculate averages and standard deviations from triplicate experiments. 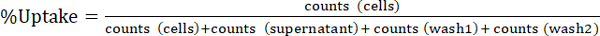          (Equ.1) NOTE: Here, reporter validation experiments are described, but non-reporter-related cellular functions (*e.g.* proliferation, cancer cell invasion, gene expression etc.) are ultimately application-specific and the responsibility of each user.

### 2. Establishment of *in vivo* tumor models

1. Use only fully characterized and validated cells for *in vivo* experiments. Check fluorescence of cells prior to administration by any suitable technique (*e.g.* fluorescence microscopy, flow cytometry) on every occasion.
2. Establish the tumor model in 5-6 weeks old young-adult female mice. Use *BALB/cAnNCrl* or *BALB/cAnN.Cg-Foxn1^nu^* (*BALB/c Nude*) mice for the 4T1 tumor model and *NOD.Cg-Prkdc^scid^ Il2rg^tm1WjI^/SzJ* (NSG) mice for the MDA-MB-231-based tumor model. Shave animals locally and use aseptic technique. Directly inject 50 µL of a suspension containing 10^6^ NIS-FP expressing cancer cells/mL into the mammary fat pad between the fourth and the fifth nipple^31^. NOTE: To improve injection accuracy, it is recommended performing the injection under general anesthesia using an inhalable anesthetic such as isoflurane (1-2 % (v/v) in O_2_). NOTE: Surgical implantation of tumor pieces from NIS-FP expressing tumors is an alternative approach to tumor model establishment^31^.
3. Check fluorescence of cells at injection sites after administration and in the early days post injection. Use a fluorescence torch and filter glasses suitable for the FP of choice.
4. Monitor tumor growth and check for any clinical signs (particularly at later time points). NOTE: For superficial tumors, *i.e.* the orthotopic breast tumors in this protocol, use calipers and the formula for the mean tumor diameter (MTD) = ½·(L+W)^32^.

### 3. Production of [^18^ F]BF _4_^-^ using an automated radiotracer synthesis (ARS) platform.

NOTE: Here, the automated [^18^F]BF_4_^-^ synthesis based on the method of ^18^F addition to boron trifluoride is described. Users of a more widely available ARS platform (see Table of Materials), can download the corresponding Extensible Markup Language (XML) file required to run the automated sequence on this platform (Supplementary File). A detailed explanation of the cassette layout shown in Figure 1 is provided in (**Table 1**) as well as a detailed description of each step in the XML sequence file (**Table 2**) to support translation to any other automated platform.

1. Set up the ARS platform as described in **Table 1** and ensure it is operational with the correct XML file loaded onto the control computer. Ensure the ARS platform is placed in a chemical hood suitable for safe work with GBq amounts of radioactivity.
2. Aspirate the cyclotron-produced [^18^F]F^-^ (typically 1.5-2 GBq in 2-7 mL [^18^O]H_2_O) via the inlet reservoir (V6).
3. Trap the radioactivity on an anion exchange resin (*e.g.* quaternary ammonium anion exchange cartridge; V5 to V4), recover the [^18^O]H_2_O in a separate vial (V1).
4. Elute the [^18^F]F^-^ (reverse elution, V5 to V4) into the reactor (V8) with 750 µL of 0.9 % (w/v) saline solution (V2), followed by 1.5 mL of acetonitrile (V16).
5. Remove the water by azeotropic evaporation under vacuum and nitrogen flow by heating at 105 °C then 120 °C for 5 min.
6. Reduce the reactor temperature to 80 °C.
7. Add 800 µL of 15-crown-5 in anhydrous acetonitrile (46 mg, 0.21 mmol) to the dry [^18^F]F^-^ through the central port of the reactor (V8).
8. Add 850 µL of BF_3_.OEt_2_ in anhydrous acetonitrile (0.16 mg, 1.13 µmol) to the reactor.
9. React for 5 min while returning to room temperature.
10. Pass the reaction mixture through an aluminum oxide cartridge (V17 to V18) to trap the trifluoroborate.
11. Return the reaction mixture to syringe S2 and dilute with water (approx. 1.6 mL).
12. Pass the reaction mixture through a second anion exchange cartridge (V19 to V20) to trap the [^18^F]BF_4_^-^ product.
13. Wash the reactor with water (approx. 5.5 mL).
14. Pass the resulting solution through the alumina and second anion exchange cartridges.
15. Rinse syringes S2 and S3 with water.
16. Wash the second anion exchange cartridge with water and dry it with nitrogen gas.
17. Elute the product (V19 to V20) with 1 mL of 0.9 % NaCl (V14) into syringe S3.
18. Transfer the product (400-500 µL) via the outlet line (V21) to a 1 mL glass collection vial. NOTE: Molar activity is an important aspect of every radiotracer. However, its routine determination is not only time-consuming but also requires significant amounts of the freshly synthesized [^18^F]BF_4_^-^, such that it becomes a limiting factor for the number of animals that can be imaged. To test reproducibility and molar activities of the resultant [^18^F]BF_4_^-^, it is recommended to schedule a few dedicated test-runs for this purpose. For more details about molar activities, please refer to the Discussion section.

### 4. *In vivo* imaging of NIS-FP expressing cells by nanoPET/CT

1. Animal preparation
  1. Anesthetize mice with 1.5-2.0 % (v/v) isoflurane in O_2_ at a flow rate of 1.0-1.5 L/min in an induction chamber. To check for sufficient anesthesia look for absence of the pedal reflex.
  2. Ensure to apply vet ointment on animal eyes to prevent dryness while under anesthesia
  3. Move the mouse onto a heating pad with the nose in an anesthetic supply mask and warm the tail (*e.g.* by dipping it into 37 °C water or using an infrared light lamp).
  4. Dilute the freshly prepared sterile-filtered [^18^F]BF_4_^-^ solution to 5 MBq per 50 µL with 0.9 % sterile saline.
  5. Using a syringe connected to a hypodermic needle (gauge 29-31), draw 100 µL of the [^18^F]BF_4_^-^ solution, measure the radioactivity in the syringe, and note the value and the time of the measurement.
  6. Intravenously administer 50 µL of the [^18^F]BF_4_^-^ solution into the pre-warmed tail vein.
  7. Measure the remaining radioactivity in the syringe and note the value and the time of the measurement. The difference between values measured at steps 4.1.7 and 4.1.5 is the injected dose (ID).
  8. Set a timer to count down from 45 min (start time of PET imaging = 0 min).
  9. Place the mouse onto the bed of the nanoPET/CT scanner and ensure the anesthetic supply is correctly re-attached.
  10. Check anesthesia remains complete by testing for absence of the pedal reflex.
  11. Ensure the mouse is positioned on the bed in the desired way, *e.g.* the 'sphinx'-like position.
  12. Install animal monitoring devices according to the manufacturer's recommendations, *e.g.* a rectal temperature probe, a probe measuring animal breathing, or electrodes for recording electrocardiograms. Check the proper function of all instruments. NOTE: For in vivo specificity tests with the NIS radiotracer [^18^F]BF_4_^-^, animals are imaged as described above, and then rested awake until the radioactivity has decayed sufficiently to be regarded as negligible, *e.g.* 48 h later when only 1.3·10^−^^6^ % residual ^18^F radioactivity will be present in the animal. In the subsequent imaging session, the competitive substrate ClO_4_^-^ is administered at a dose of 200 mg/kg 30 min prior to radiotracer administration, and imaging is performed as described above.
2. Imaging by nanoPET/CT
  1. Set the desired CT imaging parameters, *e.g.* using the nanoPET/CT 55 kVp tube voltage, set the exposure time to 1200 ms with one-degree angular stepping and 180-degree projections.
  2. Set the parameters for PET image acquisition. Use static scan PET parameters with a duration of 30 min, 1:5 coincidence mode and 400-600 keVp energy window.
  3. At countdown time = 15 min start CT image acquisition.
  4. At countdown time = 0 min start PET image acquisition.
    1. If serial animal imaging is required, let animals fully recover from anesthesia, *i.e.* regain consciousness under supervision. Subsequently, transfer them to a maintenance unit.
    2. If this is the terminal imaging session, proceed to animal euthanasia by either anesthetic overdose, rising concentration of carbon dioxide, or dislocation of the neck.

### 5. *In vivo* data analysis

1. Reconstruct the PET/CT data using a Monte Carlo-based full 3D iterative algorithm. Ensure that corrections for attenuation, dead time, and radioisotope decay are considered. For details refer to the manufacturer's instructions of the PET/CT instrument in use.
2. Check CT and PET images are correctly co-registered and save the data in a suitable exchange format such as 'Digital Imaging and Communications in Medicine' (DICOM).
3. Analyze images
  1. Load the reconstructed DICOM files into a suitable image analysis software that enables the recognition and delineation of regions of interest (ROIs) and subsequent PET signal quantification in these ROIs.
  2. Segment the ROIs using manual or adaptive thresholding to define ROIs^33^,^34^ using a suitable software package. Anatomical image information from the CT scan helps guide ROI assignment, *e.g.* superficial tumors or lung volumes.
  3. Use the analysis software as per manufacturer's instructions and ensure data are calibrated to the injected radioactivity dose and corrected for attenuation and radioactive decay.
4. Draw graphs showing data from this *in vivo* quantification. Express data as either percent injected dose/volume (%ID/mL) or standard uptake value (SUV), which is an alternative measure considering the radioactivity in the whole body of the subject.
  1. Calculate %ID/g values assuming the tissue density to be like water, i.e. ~1 g/L. It is noteworthy that this assumption can be invalid for organs with significantly different densities, such as lung or bone.
  2. Calculate different SUVs to estimate the true SUV (*e.g*. SUVmean, SUVmax); SUVmax is more reliable for small objects and is more frequently used than SUVmean^35^.

### 6. *Ex vivo* analyses

Perform the listed downstream analyses: (i) fluorescence imaging of organs containing fluorescent cancer cells (primary tumor and metastases) during animal dissection, (ii) measurement of radiotracer tissue distribution, and (iii) histologic or (iv) cytometric assessment of cancerous organs.

1. Measurement of radiotracer distribution by γ-counting (*ex vivo* biodistribution) and *ex vivo* fluorescence imaging of cancerous tissues.
  1. Measure radioactivity of the whole dead animal and note the value and the time.
  2. Dissect the animals and harvest the following tissues: lung, heart, blood (using 20 mm glass capillaries), liver, stomach, kidneys, spleen, small and large intestines, thyroid and salivary glands, a piece of muscle from the leg, and bone of the rear femurs, and relevant and dissectible lymph nodes and cancerous tissues.
  3. Measure the radioactivity of the remaining carcass first including then excluding the tail and note the values and the times of measurement. NOTE: Radioactivity in the tail can be considered stemming from radiotracer that was mis-injected and thus did not reach circulation; hence, this amount of radiotracer was not contributing to the injected dose. The tail radioactivity serves also as a retrospective parameter of injection quality.
  4. Weigh all tissues (use pre-weighed tubes).
  5. Take photographs of cancerous organs in daylight and under fluorescence light. NOTE: Use a camera stand to keep the distance between camera lens and organ constant (or use a dedicated commercial instrument for this purpose).
  6. Embed organs/tissues intended for downstream histology into OCT or immerse them in formalin for fixation. For other downstream applications sample preparation can differ.
  7. Prepare radiotracer calibration standards in duplicate, *e.g.* 0 to 1000 kBq [^18^F]BF_4_^-^. NOTE: Calibration standards are required to (i) relate the measured counts per min to radioactivity values (in kBq), and (ii) simplify decay correction; ^18^F^-^ can replace [^18^F]BF_4_^-^.
  8. Count the radioactivity of all harvested tissues using a γ-counter together with radioactivity calibration standards from step 6.1.7. Note the time of measurement. If count rates are too high (*i.e.* outside of linearity of calibration standard or indicated by too high detector dead times), re-count the samples two radiotracer half-lives later.
  9. Present data either as %ID/g or standard uptake values (SUV) (Equ.2). SUV = 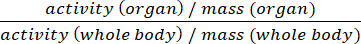       (Equ.2)
  10. Discard all harvested tissues that are not required for further downstream analyses according to local waste management rules.
2. Analyze cancerous tissues by cytometry or histology according to the user's preferences and standard protocols (as described elsewhere^3^,^6^,^28^).

## Representative Results

The first step requires genetic engineering of the cancer cells of interest. Here, the results of lentiviral transduction of metastatic murine inflammatory 4T1 breast cancer cells and human metastatic MDA-MB-231 cells with lentivirus particles carrying DNA encoding either NIS-GFP or NIS-RFP are shown. Transduction efficiencies varied between cancer cell lines (Figure 2A, left column). However, all resultant transduced cancer cell lines were selected by FACS to purity (Figure 2A, right). Confocal fluorescence microscopy (Figure 2B) demonstrated correct plasma membrane localization of NIS-FPs. NIS-FP function was quantified using NIS-afforded radiotracer uptake (Figure 2C**-2E**) and demonstrated NIS function and specificity. Notably, no significant differences between 4T1.NIS-GFP and 4T1.NIS-RFP expressing cell lines with similar NIS expression levels were found (Figure 2C).

Following full *in vitro* cell line characterization, tumor models were set up with the newly generated traceable cancer cell lines. As an example, the 4T1.NIS-GFP tumor model, a model for inflammatory breast cancer, is shown here (Figure 3). In tumor-bearing animals longitudinal whole-body PET imaging then informed on tumor progression including metastatic spread (Figure 3B). The PET radiotracer [^18^F]BF_4_^-^ was necessary for imaging and freshly produced in the morning of every PET imaging session. Synthesis of [^18^F]BF_4_^-^ was performed using the described ARS method. Typically, ~1.6 GBq ^18^F^-^ was used as input and obtained ~244 MBq [^18^F]BF_4_^-^ in 40.5±3.9 min (N=17). The product was analyzed by radio thin layer chromatography or ion chromatography and showed a radiochemical purity of 94.7±1.4 %. The radiochemical yield was 19.4±4.0 % (decay-corrected).

On day 19 after tumor inoculation, the primary tumor was clearly identified using PET, but found no metastases. Ten days later (day 29), the same tumor-bearing mice were re-imaged and distant metastasis at various locations in all animals (lung metastasis, metastasis to various inguinal and/or axillary lymph nodes) were identified. The example in Figure 3 showed extensive lung metastasis with several clearly identifiable and quantifiable nodules in the lung (Figure 3B**-3E**). Moreover, the animal presented with regional spread of the tumor into the peritoneal wall as well as metastasis to the inguinal and both axillary lymph nodes. %ID values of individual metastases in the lung (Figure 3E) differed widely, but so did the occupied volumes of the underlying metastatic nodules. In contrast, volume-normalized %ID/mL values (Figure 3E) were much more uniform. This was comprehensible for different metastases at similar development stages (*i.e.* developed between days 19 and 29; Figure 3B). In contrast, the normalized %ID/mL value for the primary tumor was lower than those for the lung metastases, which is in line with a tumor mass that had more time to progress and remodel including the influx of other cell types (stromal cells, immune cells), particularly in this model of inflammatory breast cancer.

Guided by *in vivo* images and the fluorescence of cancer cells (visible during animal dissection under fluorescence light), small deep-seated organs such as lymph nodes were reliably harvested and, at the same time, assessed for cancerous nodule content (Figure 4A). While the fluorescence signal during animal dissection was indicative of tumor cell presence, it was important to ensure this classification was accompanied by *ex vivo* radioactivity measurements of the harvested tissues. Figure 4B shows the standard uptake values (SUV) obtained for the various tissues across a cohort of three animals, all of which presented with metastasis. Endogenously NIS-expressing organs such as the thyroid and salivary glands (harvested combined) or the stomach also showed the expected high radiotracer uptake. Furthermore, this NIS-FP approach allowed straightforward cancer cell identification during histology (Figure 4C). This immunofluorescence histology example data showed tumor vascularization in the 4T1.NIS-GFP tumor model. This data also showed that the NIS-GFP reporter resided predominantly in the plasma membranes of the tumor cells also *in vivo* (Figure 4C), thereby validating the uptake results.

**Figure:**
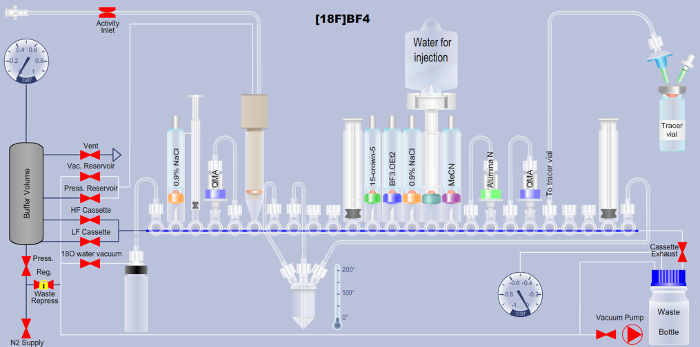

**Figure 1. Scheme detailing the set-up of the automated radiotracer synthesis platform for the production of [^18^F]BF_4_^-^ via the fluorine-18-to-boron trifluoride addition method.** Reagent names are printed onto the respective tubes in the scheme. QMA is the abbreviation for quaternary ammonium anion exchange, and indicates the used solid-phase chromatographic separation material. Additional details are available in Tables 1 and 2. Please click here to view a larger version of this figure.

**Figure:**
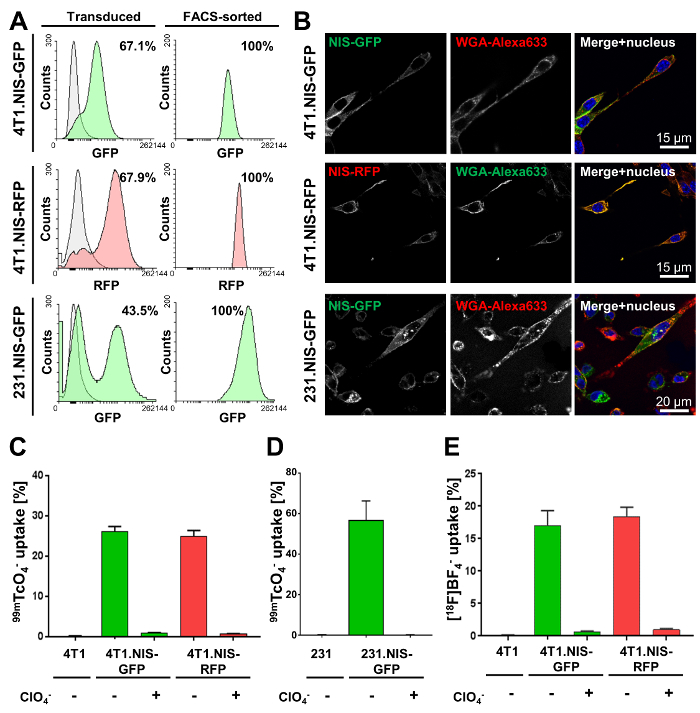

**Figure 2. Typical characterization results of cancer cell lines stably expressing NIS-GFP or NIS-RFP. (A)** The indicated cell lines were made using lentiviruses transferring either NIS-GFP or NIS-RFP. The left column shows the transduced population (green or red fluorescent) as compared to the respective parental cells (grey; 4T1 and MDA-MB-231 cells, respectively). Percentages show transduction efficiencies as determined by flow cytometry. The right column shows the results of flow cytometric analyses after FACS purification of the mixed populations in the left column. All cell lines were found to be >99 % pure for indicated NIS-expressing cells (by flow cytometry). **(B)** Confocal fluorescence microscopy of purified cell lines shows plasma membrane localization of NIS-GFP or NIS-RFP in the respective cell lines. WGA-Alexa633 was used as a plasma membrane marker. **(C, D)** Functional validation of NIS-FP protein expressed in the indicated newly generated cancer cell lines. NIS function was measured using the radiotracer ^99m^TcO_4_^-^ (50 kBq per million cells). As controls, parental cells were used as well as fusion reporter expressing cells that were treated with the NIS co-substrate perchlorate before and during the assay (specificity control). Results clearly demonstrate NIS-FP function and specificity in all cell lines. **(E)** Functional validation of 4T1.NIS-FP cell lines using [^18^F]BF_4_^-^ as a radiotracer for NIS. All other conditions were identical to (C). Importantly, very similar relative uptake results were obtained for both 4T1-derived cell lines with both radiotracers (Figure 2C and E), thereby justifying the interchangeable use of both for *in vitro* functional characterization of NIS-FP expressing cell lines. Please click here to view a larger version of this figure.

**Figure:**
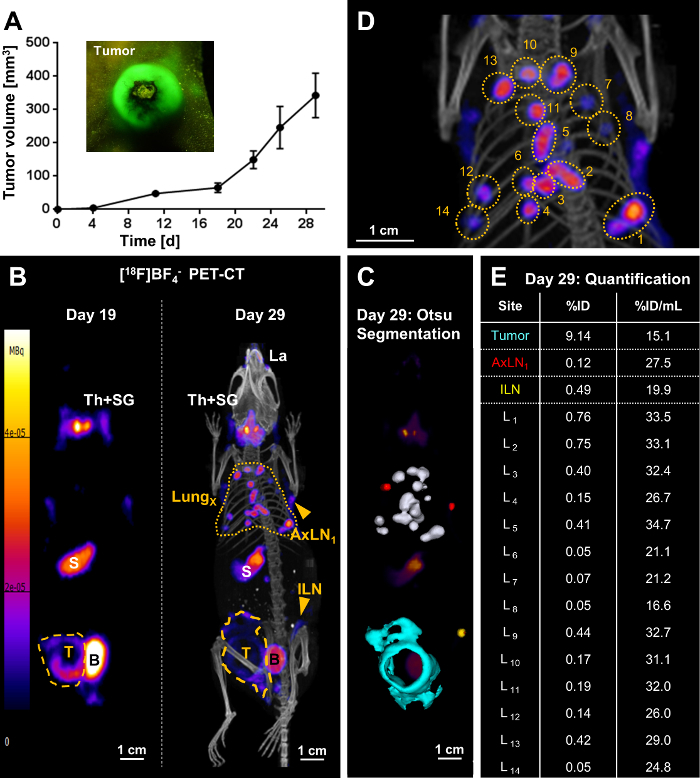

**Figure 3. Representative result of metastasis tracking by [^18^F]BF_4_^-^-PET/CT imaging in a mouse bearing a 4T1.NIS-GFP tumor.(A)** One million 4T1.NIS-GFP cells were injected into the mammary fat pads of 5-6 weeks old BALB/c CanN.Cg-Foxn1^nu^/Crl mice and tumor growth was followed over time using calipers. Owing to the GFP fluorescence of the cancer cells, crude visual identification/growth assessment was also possible using a fluorescence torch and suitable filter glasses (see inset). **(B/left)** On day 19 post tumor inoculation, the primary tumor (yellow dashed line) was clearly identified but no metastasis. The image presented is a maximum intensity projection (MIP) of the PET image. Endogenous NIS signals (white descriptors) were also recorded, *i.e.* the thyroid and salivary glands (Th+SG), the stomach (S), and, at very low levels, some parts of the mammary and lachrymal glands. The bladder (B) signal stems from tracer excretion. **(B/right)** On day 29 post tumor inoculation, metastasis was clearly identified: multiple metastases in the lung (yellow dotted line) as well as metastatic lymph nodes (ILN, AxLN; yellow arrowheads). The image presented is a MIP of the PET/CT image. The primary tumor (yellow dashed line) grew not only in a globular shape at this time point, but also had invaded into the peritoneal wall. **(C)** A 3D implementation of the Otsu thresholding technique enabled 3D surface rendering of the cancerous tissues; these are superimposed onto a PET MIP. Lung metastases are shown in white, metastatic axillary lymph nodes in red, the metastatic inguinal lymph node in yellow, and the primary tumor that invaded into the peritoneal wall in turquoise. **(D)** A blow-up image of the PET/CT MIP in (B/right) to indicate individual lung metastases. **(E)** Radiotracer uptake into cancerous tissues was quantified from 3D images (%ID) and normalized by their respective volumes (%ID/mL). Individual lung metastases correspond to the numbering in (D). Please click here to view a larger version of this figure.

**Figure:**
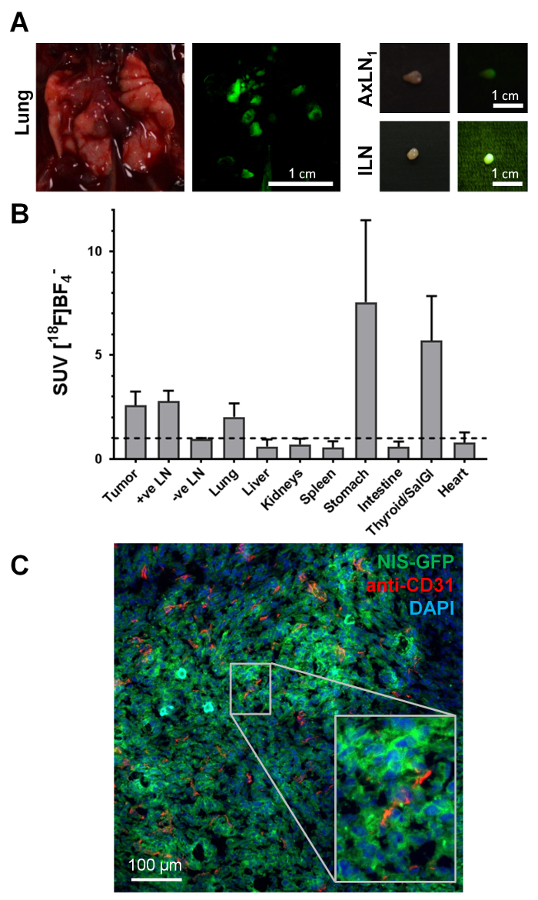

**Figure 4. Typical examples of *ex vivo* data accessible from NIS-FP tumor-bearing mice. (A)** During tissue harvesting for downstream analyses, the fluorescent properties of the NIS-FP expressing tumor cells served as an indicator guiding animal dissection. As exemplars, tissues from the animal in Figure 3, *i.e.* the lung with several metastatic lesions and two positive lymph nodes are shown. Daylight photography as well as fluorescence images are shown. The fluorescence images were taken with the same camera as the daylight images but under blue light excitation (450±10 nm bandpass filter) with a green emission filter (530±30 nm bandpass filter) placed in front of the camera lens. **(B)** Distribution of radiotracer in different organs ('biodistribution') of animals with 4T1.NIS-GFP tumors (N=3; day 29 post tumor inoculation; 5 MBq [^18^F]BF_4_^-^). Standard uptake values (SUV) were calculated and values >1 indicate specific accumulation of radiotracer in the respective organs. The data show specific radiotracer uptake in cancerous tissues, *i.e.* primary tumor, metastatic lymph nodes (as identified by imaging and dissection under fluorescence light), lung (was dissected as a whole without separating individual metastases), as well as organs endogenously expressing NIS, *i.e.* thyroid and salivary glands and stomach. **(C)** Immunofluorescence histology of the primary tumor from the same mouse as shown in Figure 3. The primary tumor was harvested, embedded in OCT and frozen before being sectioned (10 µm) and processed for staining. NIS-GFP expressing cancer cells were directly identified without the need for antibody staining. Blood vessels were stained with a rabbit antibody against mouse PECAM-1/CD31 (2 µg/mL) and a Cy5-conjugated goat anti-rabbit secondary antibody. Nuclei were stained with 2'-(4-ethoxyphenyl)-5-(4-methyl-1-piperazinyl)-2,5'-Bi-1H-benzimidazole (1 µg/mL) and the sample mounted in poly(vinyl alcohol - vinyl acetate) containing 2.5 % (w/v) Dabco as an antifade. Confocal images were obtained using a confocal microscope with settings appropriate for 2'-(4-ethoxyphenyl)-5-(4-methyl-1-piperazinyl)-2,5'-Bi-1H-benzimidazole, GFP and Cy5. These example data clearly show that the 4T1.NIS-GFP tumor is vascularized but also that vascularization differs in its extent (*cf.* top left with bottom middle). It also shows that the NIS-GFP reporter predominantly resides in the plasma membranes of the tumor cells *in vivo* (inset), thereby validating the *in vitro* uptake results. Please click here to view a larger version of this figure.

**Table:** 

**FASTlab manifold valve**	Reagent, solvent, cartridge or tubing*	**Details**
**V1**	Silicone tubing to [^18^O]H_2_O waste bottle	14 cm
**V2**	0.9% NaCl solution, 750 µL	11 mm vial
**V3**	Syringe S1	1 mL
**V4**	anion exchange cartridge C1, pre-conditioned with 1M NaCl (10 mL) and H_2_O (10 mL)	*e.g.* Sep-Pak Accell Plus QMA Plus Light (Waters, cat. No. WAT023525)
**V5**	Silicone tubing to anion exchange cartridge C1	14 cm
**V6**	[^18^O]H_2_O/^18^F inlet reservoir	Max 5 mL
**V7**	Silicone tubing to reactor vessel (left side; gas inlet)	14 cm
**V8**	Silicone tubing to reactor vessel (central port; liquid inlet/outlet)	14 cm
**V9**	Closed	
**V10**	Closed	
**V11**	Syringe S2	5 mL
**V12**	15-crown-5, 46 mg in 800 µL MeCN	11 mm vial
**V13**	Trifluoroborate diethyl etherate, 0.14 µL in 850 µL MeCN (dilute 14 µL of BF_3_.OEt_2_ with 1 mL MeCN. Dilute 10 µL of this solution to 850 µL with MeCN).	13 mm vial
**V14**	0.9% NaCl solution, 1 mL	13 mm vial
**V15**	Water bag spike	
**V16**	Acetonitrile (MeCN), 1.5 mL	13 mm vial
**V17**	Silicone tubing to Alumina neutral cartridge C2	14 cm
**V18**	Alumina neutral cartridge C2, pre-conditioned with H_2_O (10 mL), acetone (10 mL) and air (20 mL)	*e.g.* Sep-Pak Alumina N Plus Light (Waters, cat. No. WAT023561)
**V19**	Silicone tubing to anion exchange cartridge C3	14 cm
**V20**	anion exchange cartridge C3, pre-conditioned with 1 M NaCl (10 mL) and H_2_O (10 mL)	*e.g.* Sep-Pak Accell Plus QMA Plus Light (Waters, cat. No. WAT023525)
**V21**	Silicone tubing to collection vial	40 cm
**V22**	Closed	
**V23**	Closed	
**V24**	Syringe S3	5 mL
**V25**	Silicone tubing to reactor vessel (right side; vacuum port)	40 cm
*NOTE: Because of the plastic spikes, the dead volume for 11 mm vials and 13 mm vials is approximately 0.35 mL and 0.4 mL, respectively. Therefore, the actual amounts of reagents transferred to the reactor are slightly different. All quantities indicated in this method refer to the actual amounts introduced in each reagent vial.

**Table 1. Description of the cassette layout for the automated [^18^F]BF_4_^-^ synthesis via the fluorine-18-to-boron trifluoride addition method** (*cf.* Figure 1)**.**

**Table:** 

**Sequence steps**	**Comment**
[1-2]	Pressurize the system and flush the manifold with N_2_
[3-15]	Rinse syringe S3 twice with H_2_O (V15), flush the manifold with N_2_
[16-23]	Pressurize reagent vials in positions V16, V14, V13 and V12, flushing the manifold with N_2_ between each vial
[24-26]	Open the activity inlet (V6)
	Connect the vial containing ^18^F. If the total volume is > 5 mL, only insert the needle halfway into the vial before continuing.
[27-39]	Close the activity inlet (V6), trap ^18^F in QMA cartridge C1 (V5), collect the [^18^O]H_2_O in the waste bottle (V1). If the total volume is > 5 mL, pause the sequence at step 37, return to step 26, fully insert the needle into the vial containing ^18^F, and resume the process.
[40]	Close the [^18^O]H_2_O waste bottle (V1), flush the manifold with N_2_
[41]	Pressurize the eluent vial in position V2
[42-44]	Open reactor valve V8, aspirate eluent from V2 into syringe S1
[45-50]	Elute QMA cartridge C1 into reactor (V8) using saline from syringe S1, set the reactor temperature to 90 °C
[51]	Flush QMA cartridge C1 with N_2_ and increase the reactor temperature to 105 °C
[52-53]	Draw acetonitrile from V16 into syringe S2
[54-57]	Transfer acetonitrile from syringe S2 to the reactor (V8)
[58-60]	Heat the reactor at 120 °C for 5 min. Evaporate the solvent with a flow of N_2_ to the reactor (V7).
[61-65]	Set the temperature to 105 °C, dry syringe S1 with N_2_
[66-69]	Draw the 15-crown-5 solution from V13 into syringe S2, increase the reactor temperature to 120 °C
[70-71]	Reduce the temperature to 105 °C, flush the manifold with N_2_
[72]	Cool down the reactor (set the temperature to 40 °C) for 5 min
[73-78]	Set the reactor temperature to 80 °C, transfer the 15-crown-5 solution from syringe S2 to the reactor (V8)
[79-81]	Draw the BF_3_.OEt_2_ solution from V14 into syringe S2
[82-87]	Transfer the BF_3_.OEt_2_ solution from syringe S2 to the reactor (V8), flush the reactor line with N_2_
[88]	Flush the manifold with N_2_
[89]	React for 5 min, let the temperature return to RT
[90-95]	Transfer the reaction mixture (V8) to syringe S2
[96-104]	Pass the reaction mixture through Alumina N cartridge C2, into syringe S3
[105]	Flush the manifold with N_2_
[106-109]	Return the reaction mixture to syringe S2
[110-112]	Empty syringe S3, draw H_2_O (V15) into syringe S2 to dilute the reaction mixture
[113-115]	Load the reaction mixture onto QMA cartridge C3
[116-118]	Draw H_2_O (V15) into syringe S2
[119-124]	Rinse the reactor (V8) with H_2_O from syringe S2, aspirate the washings into syringe S2
[125-128]	Pass the washings through cartridges C2 and C3
[129-130]	Dry the cartridges and the manifold with N_2_
[131-136]	Wash syringe S1 with H_2_O (V15)
[137-142]	Wash syringe S2 with H_2_O (V15)
[143]	Flush the manifold with N_2_
[144-147]	Draw H_2_O (V15) into syringe S2
[148-151]	Flush QMA cartridge C3 with H_2_O from syringe S2
[152-153]	Dry QMA cartridge C3 with N_2_ and flush the manifold with N_2_
[154-157]	Elute QMA cartridge C3 with 0.9% NaCl (V14) into syringe S3
[158-161]	Transfer the product from syringe S3 to the collection vial (V21)
[162-163]	Flush QMA cartridge C3 with N_2_ to the collection vial (V21)
[164-166]	Flush the manifold with N_2_
[167-170]	Flush cartridges C2 and C3 (to waste bottle) and the manifold with N_2_
[171]	Flush the collection tubing (V21) with N_2_

**Table 2. Description of steps in the XML sequence file.**

## Discussion

The first step to render cancer cells traceable *in vivo* by this method requires engineering them to express the NIS-FP fusion reporter. The choice of the fluorescent protein in the fusion reporter is critical as oligomerizing fluorescent proteins can lead to artificial reporter clustering, thereby negatively affecting its function. We have had success with proven monomeric fluorescent proteins such as mEGFP (with the monomerizing mutation A206K^36^,^37^), mTagRFP, or mCherry. NIS can either be of human or mouse origin (hNIS or msNIS) depending on the purpose of the experiment and the cancer model. Transduction efficiencies generally vary between different cancer cell lines. However, generated cancer cell lines are subsequently purified by FACS in this protocol, thereby reducing the need for optimizing transduction conditions. Transduction with high multiplicity of infection is not always advisable as multiple construct integration into the genome is likely to result not only in higher construct expression but also in more unwanted/unregulated genome modification. Therefore, it is important to let polyclonal transduced cells grow to stability of expression (monitored by flow cytometry) and avoid sorting the brightest clones only by FACS. It also renders functional validation of non-reporter features crucial before these cells should be used for *in vivo* experiments. A recently developed alternative to viral gene delivery is gene editing technology^38^, which offers more specific control over viral integration sites. Expression analysis by flow cytometry and immunoblotting is important. Flow cytometry allows acquisition of population-based single cell data, for example to examine whether there is any drift in reporter expression levels over time. It relies on the FP moiety only, unless cells are also stained with an antibody directed against surface or total NIS. Flow cytometry does not inform on fusion reporter integrity. In contrast, immunoblotting reports on the integrity of the fusion reporter. The molecular weight of NIS and the FP must be added to determine the expected molecular weight of the chosen NIS-FP. Confocal fluorescence microscopy demonstrated fusion reporter colocalization with the plasma-membrane marker wheat germ agglutinin in all newly made cell lines. This was the expected cellular location for most of the protein and indicated a go-ahead milestone for subsequent functional validation. If minimal/no NIS-FP was found on the plasma membrane (*e.g.* only in internal cellular compartments), this would indicate a cell biological issue with the fusion reporter in this cell line, or a potential mutation of the fusion reporter affecting its intracellular trafficking. It is noteworthy that we have not observed such a case in any of the cancer cells we tested so far, which included: A375P, A375M2, SK-Mel28, WM983A/B (human melanoma); MCF-7, MDA-MB-231, MDA-MB-436 (human breast cancer); NCI-H1975 (human lung cancer); SK-Hep1 (human liver cancer); 4T1, 4T1.2, 66cl4, 67NR, FARN168 (murine inflammatory breast cancer); B16F0, B16F3, B16F10 (murine melanoma); MTLn3 (rat breast adenocarcinoma).

NIS function must be measured using uptake assays with radioactive NIS substrates. Due to the SPECT radiotracer ^99m^TcO_4_^-^ being generator-produced and therefore widely available in hospitals without the need for any radiotracer synthesis as well as having a more convenient longer half-life (6.01 h for ^99m^Tc as compared to 110 min for ^18^F), we used this NIS substrate for routine functional validation of new NIS-FP expressing cell lines. Pre-blocking of NIS-expressing cells with the NIS co-substrate sodium perchlorate resulted in the expected reduction/abolishment of radiotracer uptake, thereby demonstrating specificity of radiotracer uptake. This NIS specificity test is a critical validation step. If a NIS specificity experiment would not result in reduced radiotracer uptake comparable to the respective parental cells, either a technical error during the experiment has occurred, or the radiotracer uptake was not due to NIS. It is also possible that sodium perchlorate pre-blocking reduces radiotracer uptake in a parental cell line; this would identify cell lines with endogenous functional NIS expression (*e.g.* stimulated thyroid cells^6^).

A crucial advantage of this imaging protocol is that information is collected in 3D and over time. This allows the comparison of images from the same animal over time, thereby providing paired data and thus overcoming the issues caused by inter-animal variability. This contrasts with most non-imaging related metastasis assessment methods that are based on sacrificing different animals at different time points. In **Figure 3B** it is evident how metastatic spread and outgrowth progressed over time in an individual animal. The signals detected by PET/CT imaging are fundamentally caused by NIS expression. This includes all signals from exogenously NIS-expressing cancer cells as well as all organs endogenously expressing NIS. Typical endogenous NIS signals are found in thyroid and salivary glands, the stomach, and, at low levels in some parts of the mammary and lachrymal glands. In addition to endogenous NIS expression, the NIS radiotracer [^18^F]BF_4_^-^ is also excreted via the kidneys, thereby explaining radiotracer uptake in urine-filled bladders. Kidney uptake is no longer detectable at the imaging time point recommended in this protocol (45 min post radiotracer injection^6^). If signals from urine-filled bladders should lead to signal-to-background issues, the bladder can be mechanically emptied under anesthesia before imaging. Importantly, the endogenous signals can vary between animal strains. It is also noteworthy that endogenous NIS expression in the mammary glands can be higher under lactating conditions^10^. In the presented case and in the cases of those metastatic cell lines successfully characterized before (*cf.* list above), we did not find endogenous NIS expression to significantly interfere with metastasis detection. It is noteworthy, that [^18^F]BF_4_^-^ remains more available for uptake into cancerous tissues as compared to iodide, because iodide is metabolized into thyroid hormones^6^. This phenomenon might also contribute to larger amounts of radioiodide in the blood stream as compared to [^18^F]BF_4_^- 6^. For different applications (cancer cell tracking in other cancers or non-cancer cell tracking applications), this might differ, and it is therefore recommended to assess whether endogenous NIS expression is likely to cause signal-to-background issues through preliminary experiments. An important aspect in preclinical imaging is the molar activity of the radiotracer. The method described here uses ~1.5 GBq ^18^F^-^ as starting material^14^ and has been shown to produce molar activities significantly above the previously reported substitution method^12^. [^18^F]BF_4_^-^ produced at molar activities ≤1 GBq/µmol^12^ can lead to reduced uptake in NIS-expressing tissues. This is of particular importance when the injected amount of radioactivity per kilogram is high, *i.e.* when small animals such as mice are imaged^39^; it is less important in the human setting^40^. High molar activities are therefore imperative for high-quality preclinical PET imaging. Molar activities obtained by the boron trifluoride addition method^14^, which is shown in its automated form in this protocol, overcome this issue. Furthermore, it is noteworthy that the presented protocol for [^18^F]BF_4_^-^ synthesis is not compliant with good manufacturing practice (GMP) and therefore unsuitable for use in human clinical trials in this form. A GMP protocol (via the ^18^F substitution method to radiolabel BF_4_^-^) is available elsewhere^40^.

PET/CT imaging allows the visualization of radiotracer uptake, which is indicative of NIS-mediated radiotracer uptake stemming from NIS-FP expressing cancer cells. More importantly, the associated PET signals can be quantified. It is necessary to apply reliable thresholding procedures to ensure a consistent and unbiased differentiation of relevant signals from any potential background. As the background varies in different locations *in vivo*, it is important to consider local/regional thresholding and segmentation. One such method was developed by and named after Otsu^34^, and its 3D implementation is employed for 3D rendering of the primary tumor and metastases in this protocol. Generally, the image seen by the observer visually corresponds best to the quantified %injected dose (%ID) values. As for image-based quantification, it is also important to normalize the measured radioactivity values of the different tissues to their volumes. There are two predominantly used ways of expressing normalized results, (i) %ID per volume (*e.g.* %ID/mL), and (ii) standard uptake value (SUV^35^). They differ in that %ID/mL takes into account the individual volume only, while SUV is a measure that is relative to the average radioactivity across the whole animal. It is also important to note that NIS imaging renders the live tumor volume (LTV) accessible, because dead/dying cells not synthesizing ATP can no longer import radiotracer^10^. This explains the large low-signal area within the primary tumor ("donut shaped" tumor) indicating areas of tumor cell death/necrosis. Importantly, LTV was a much more reliable measure of tumor burden as compared to the crude tumor volume accessible by caliper measurements (which does not take into account viability and assesses only superficial tumor regions).

A major advantage of this dual-mode tracking strategy is evident when harvesting tissues after animal culling. Guided by *in vivo* images and assisted by fluorescent cancer cells during animal dissection, small and deep-seated organs/metastases can also be reliably harvested. Frozen tissue preservation/sectioning methodology enables the direct fluorescence imaging of GFP without the need for staining with an anti-GFP antibody, but at the expense of reduced structural tissue preservation as compared to formalin-fixed paraffin-embedded methodology (FFPE). The latter critically requires also anti-FP staining, because the FFPE method is incompatible with intact preservation of fluorescent proteins (due to fixation/dehydration/rehydration). While the fluorescence signal is indicative of tumor cell presence, it is important to ensure this classification is confirmed by *ex vivo* radioactivity measurements of the harvested tissues ('biodistribution'). *Ex vivo* radioactivity measurements are more sensitive than visual detection of fluorescence, hence can allow the identification of cancer cell-dependent signals that would otherwise remain undetected. In the case of a terminal imaging session, it is critical to accurately note the injected radiotracer amounts as well as the times of radiotracer radioactivity measurements, animal injection, animal culling, and calibrated scintillation counter measurements of harvested tissues. This is crucial to ensure correction for radiotracer decay and thereby enable reliable biodistribution analysis.

PET/CT imaging enables repeated non-invasive 3D quantification of tumor progression including the assessment of metastatic spread on a whole-body level. This feature is a significant advantage over conventional methods, which often rely on large cohorts of animals that are euthanized for the assessment of tumor progression at varying time points. The advantages of this imaging-based approach are: (i) highly sensitive non-invasive 3D *in vivo* quantification, (ii) a significant reduction in animal numbers due to the possibility of repeat imaging, (iii) the acquisition of longitudinal paired data from subsequent imaging sessions improving statistics by excluding inter-animal variability, which in turn further reduces animal numbers, (iv) automated production of [^18^F]BF_4_^-^ at high specific activities, and (v) the intrinsic option for *ex vivo* confirmation in tissues by fluorescence methodologies such as microscopy or cytometry.

*In vivo* cell tracking is a growing field. It has been fueled by recent advancements in imaging technology, which resulted in enhanced resolution, detection limits and multiplex capability (via multi-modal imaging). In this protocol, we apply this concept to track tumor progression including spontaneous cancer cell metastasis in 3D by repeat imaging. Applications include studies aimed at unraveling the mechanisms of spontaneous cancer cell metastasis. For example, traceable tumor cells could be used to study the impact of different immune cell components (as present/functional in animal strains of different levels of immunocompromisation) on the metastatic process. Similarly, the impact of individual genes, either in the animal strain or the cancer cell line, could be studied. Furthermore, the presented protocol could be used to assess/validate the efficacy of specific drugs or therapeutic concepts on tumor progression. Importantly, this reporter gene:radiotracer pair for PET imaging (NIS:[^18^F]BF_4_^-^) could also be used for different cell tracking applications. For example, several cell therapies are currently emerging as promising therapeutic approaches. This includes cellular therapeutics for cancer treatment^41^ but also in transplantation^42^ and regenerative medicine^43^,^44^ settings. Whole-body *in vivo* cell tracking is becoming increasingly important for the development and clinical translation of cellular therapeutics, for example, for evaluating safety and for therapy monitoring.

## Disclosures

The authors declare that they have no competing financial interests.
